# The potential for coral reef establishment through free-living stabilization

**DOI:** 10.1038/s41598-017-13668-7

**Published:** 2017-10-17

**Authors:** S. J. Hennige, H. L. Burdett, G. Perna, A. W. Tudhope, N. A. Kamenos

**Affiliations:** 1 School of GeoSciences, University of Edinburgh, James Hutton Road, Edinburgh, EH9 3FE UK; 2 Lyell Centre for Earth and Marine Science and Technology, Edinburgh, EH14 4AP UK; 3 School of Energy, Geoscience, Infrastructure and Society, Heriot-Watt University, EH14 4AS Edinburgh, UK; 4 School of Geographical and Earth Sciences, University of Glasgow, Glasgow, G12 8QQ UK; 5 Red Sea Research Center, King Abdullah University of Science and Technology (KAUST), Thuwal, Saudi Arabia

## Abstract

Corals thrive in a variety of environments, from low wave and tidal energy lagoons, to high energy tidal reef flats, but remain dependent upon suitable substrate. Herein we reviewed the phenomenon of free-living corals (coralliths), examined whether they have the capacity to create their own stable habitat in otherwise uninhabitable, poor substrate environments through ‘free-living stabilization’, and explore their potential ecological role on coral reefs. This stabilization could be achieved by coral settlement and survival on mobile substrate, with subsequent growth into free-living coralliths until a critical mass is reached that prevents further movement. This allows for secondary reef colonization by other coral species. To preliminarily test this hypothesis we provide evidence that the potential to support secondary coral colonisation increases with corallith size. Due to the limited diversity of corallith species observed here and in the literature, and the lack of physiological differences exhibited by coralliths here to static controls, it seems likely that only a small selection of coral species have the ability to form coralliths, and the potential to create their own stable habitat.

## Introduction

Individual corals and patch reefs can be found in a variety of environments, including those dominated by unsuitable substrate such as sand or mobile rubble^1,2^. Although prograding into such habitats is possible across millennial time scales (where corals grow, break, and re-grow from the reef edge into muddy or soft sediments)^3^, herein we explore whether coral survival and reef formation in otherwise unsuitable environments can be achieved via the creation of stable habitat by free-living corals. These ‘coralliths’^4^ are documented in reefs in all major reef systems worldwide^4–14^, and have occurred since at least the middle Pleistocene^7^. They are characterized by an unusual ability to survive mechanical stress from movement and abrasion^4^, and they settle on unstable substrate such as coral rubble^10^. *Porites* is one of the most common corallith genera, and this genus is known to be relatively resilient to environmental pressures^15,16^.

The idea of free-living organisms stabilizing their own habitat and supporting high biodiversity is well-established in the algal field, and the individual thalli of certain algal species may interlock creating a three-dimensional stable ‘reef’ on an otherwise homogenous, sandy substrate^17,18^. However, commonly known free-living coral species such as *Fungia* spp. and *Heliofungia* spp. do not interlock, and even at their maximum size they are still considered mobile. Thus, they can only provide a stable substrate for coral settlement once dead and ‘cemented’ onto stable substrate by crustose coralline algae (CCA). Conversely, free-living massive coral species (coralliths) have no upper size limit, and can become stable while still alive.

Given the variety of species and sizes of documented coralliths, we refer to coralliths here as living coral colonies that are not attached to the substrate (and hence can be moved by waves, currents and grazing), and constitute genera which are not typically considered to be ‘free-living’ (as opposed to *Fungia* spp). Once coralliths reach a stable size and support secondary colonization, they are no longer ‘free-living.’ However, large storm events could still cause movement and relocation of these coralliths. For the purposes of this study, coralliths that have ceased to move in response to wave and current action are termed ‘stable coralliths.’

**Figure 1:**
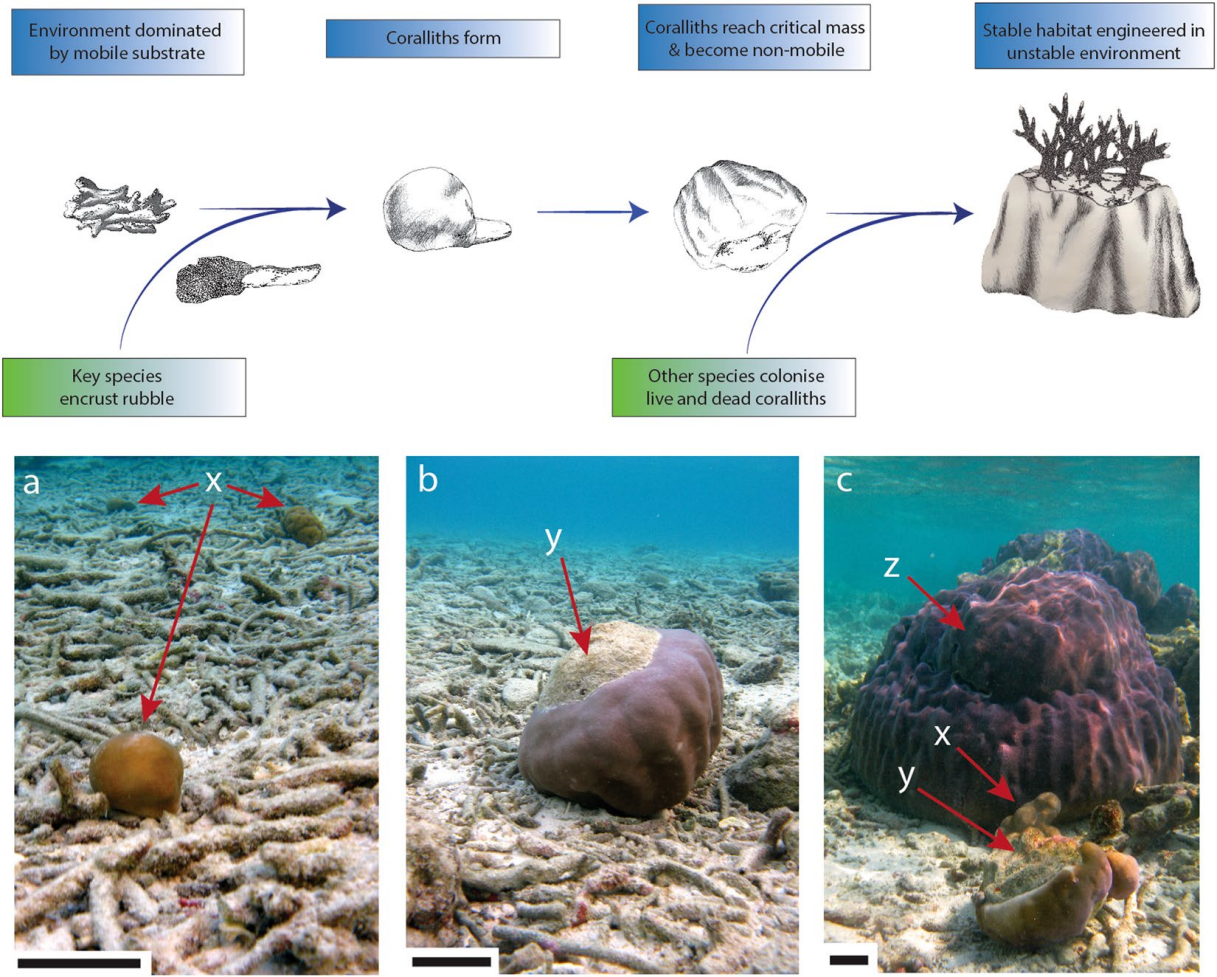
The free-living stabilization hypothesis. Only certain massive species can survive settlement on mobile rubble. These species form free-living ‘coralliths’ (**a**), which increase in size until a critical threshold is reached and no further movement occurs, creating a stable substrate for the settlement of other species (**b**,**c**). X indicates free-living *P. lutea* coralliths covered completely by tissue. Y provides examples of how a free-living *P. lutea* corallith of near-critical mass has been turned over, revealing bare substrate suitable for settlement. Z is an example of a large, static coral with suspected corallith origins (given lack of stable substrate in area) that has reached critical mass and is immobile. Scale bars are 10 cm. Illustrations by L. McWhinnie.

Herein, we examined whether coralliths can (1) grow to ‘stable’ dimensions in a reef environment dominated with rubble, and (2) act as stable substrate for further coral colonization. We hypothesized that such coralliths may differ physiologically from corals embedded in the reef framework to reflect that they will be subject to rapid changes in light availability from sudden movement, and as such may employ different protective mechanisms to prevent (photo) damage, including different pigments and photosynthetic mechanisms. We further hypothesized that the ensuing dataset could be used to test the ‘free-living stabilization hypothesis’ (Fig. 1), in which free-living coral species establish stable substrate for coral colonization by settling and surviving on a mobile substrate as coralliths. They subsequently grow until a point is reached at which movement ceases and stable habitat is formed; other corals can then colonise the corallith.

## Materials and Methods

### Location and ecology

The seaward (west) side of Vavvaru Island, Lhaviyani Atoll (5.4184°N, 73.3548°E), Maldives was used as the study location during March 2015; it is separated from the nearest Maldivian atoll by ~31 km, and is a typically seaward reef system of the area. Triplicate 25 m × 2 m belt transects were conducted parallel to the shore on the reef flat at near- (70 m from the shore), mid- (140 m), and far-shore (210 m) locations; all three locations were at identical depths (1 m). Beyond the reef flat and at the reef wall, depth dropped to ~ 30 m. Reef flat diel temperatures ranged from 27.1–31.4 °C, with an average of 29.0 °C (Table 1). Maximum photosynthetically active radiation (PAR, using an Odyssey PAR logger [Dataflow Systems Ltd]), calibrated against a LICOR) averaged 1250 µmol m^2^ s^−1^ at 1 m depth. Water chemistry was measured from nine random water samples taken from across the reef flat from where the sites were located (Table 1). Total alkalinity and dissolved inorganic carbon were analyzed as described below. Seabed substrates (not including live coral) varied between sites; the near-shore site featured predominantly branching rubble and sand while the mid-shore site was similar, though with numerous, dead massive corals. The far-shore site’s benthos was dominated by branching coral rubble, sand and larger, dead massive corals. Along each transect, the number and size (maximum dimension) of all coralliths, and total number of non-free living coral individuals were non-invasively recorded. To determine whether large, static coral colonies were mobile and had originated as coralliths, the base substrate was examined to determine whether it was part of a larger bedrock feature. Where possible, this was tested (in instances where this would not cause damage to the corals or associated organisms) by gently pushing the corallith from one side to see if there was any movement. Following ecological surveys, 10 free-living *Porites lutea* coralliths of ~10 cm diameter from an area between near- and mid-shore transect sites abundant in suitable coralliths and static controls, were sampled on all sides for physiological measurements to provide life history ‘context’ of coralliths compared to static corals (for specific details see below). As static controls, 5 small *P. lutea* colonies (similar in size to the coralliths and assumed to be approximately the same age), were carefully removed from static substrate (CCA cemented reef framework) in the same localized area, between near- and mid-shore transect sites. Following physiological experimentation *in situ* and in the laboratory, all specimens were returned alive to their site of collection.

**Table 1:** Environmental parameters of the reef flat from 1 m depth where the 3 survey sites where located.

Temperature °C	Maximum Photosynthetically Active Radiation (PAR) µmol m^2^ s^−1^	Dissolved O_2_ (mmol)	Total Alkalinity (µmol Kg^−1^)	Dissolved Inorganic Carbon (µmol Kg^−1^)	pH_T_	Omega aragonite (Ω_Ar_)
29.04 (0.03)	1250	0.14 (0.004)	2302 (20.2)	1991 (11.6)	7.99 (0.04)	3.59 (0.27)

Chemistry samples are from nine samples taken at random over the reef flat. Values are mean ± (standard error) unless otherwise indicated.

### Physiology

To assess whether coralliths are physiologically specialized for a mobile life in comparison to static conspecifics, standard physiological parameters including photosynthetic efficiency, calcification, oxygen production, and reflectance profiles (which are a function of pigmentation, structure and morphology) were examined.

#### PAM fluorometry

Rapid light curves (RLC) were conducted on the upper and lower (orientation as found) surfaces of all *P. lutea* coralliths (n = 10) and control static colonies (n = 5) in mid-afternoon (15:00–16:00) using a Diving - Pulse Amplitude Modulated fluorometer (Walz, GMBH), following Hennige *et al*.^19^.

#### Incubations

Calcification rates and oxygen production (net photosynthesis) were assessed through controlled incubations in sealed 650 ml chambers *in situ* during the afternoon (15:00–16:00) over two sequential days, for coralliths (n = 10), and controls (n = 5) respectively. Water samples were taken at the beginning and end of the 30 min incubation, analysed on-site for dissolved oxygen concentration with a YSI Pro2020 O_2_ meter, and poisoned on-site with HgCl_2_ for total alkalinity and dissolved inorganic carbon analysis^20^. Total alkalinity was determined via semi-automated titration (Metrohm 848 Titrino plus)^20^ combined with spectrometric analysis using bromocresol indicator (Smart pH cuvettes, Ocean Optic Ltd and Hach DR 5000™ UV–Vis spectrophotometer; analytical precision: ±11 μmol kg^−1^)^21^, and dissolved inorganic carbon using an Automated Infra Red Inorganic Carbon Analyzer (AIRICA, Marianda instruments analytical precision: ±2 μmol kg^−1^). Certified seawater reference materials were used for oceanic CO_2_ (Batch 141, Scripps Institution of Oceanography, University of California, San Diego) as standards to quantify analytical precision^20^.

Calcification rates were calculated using the alkalinity anomaly technique^22^. Surface areas of the coralliths using the tinfoil method, and images were analysed with Image Tool as outlined in Hennige *et al*.^23^. The volume of each coral was calculated by displacement in seawater.

#### Reflectance

Immediately following *in situ* experimentation, samples were returned to the laboratory for reflectance measurements. Single measurements were taken from each sample on the upper and lower surfaces according to the *in situ* orientation at time of sampling (measurements on upper surface only for static control corals), following methodology described in Burdett *et al*.^24^ with an Ocean Optics USB 2000+ spectrometer.

#### Sectioning

Coralliths (n = 3) were covered in EpoHeat epoxy resin (Buehler) and placed in a vacuum oven at 60 °C for 4 hours to harden epoxy before single cross- and longitudinal-sectioning. Flat sections were polished and imaged using a flatbed scanner.

#### Statistics

For data that met normal distribution assumptions and equal variances, analyses of variance were used (one-way ANOVA). Where normality assumptions were not met, Kruskal–Wallis tests were used with Dunn’s Multiple comparison. To assess potential correlations between photosynthetic parameters and coral abundances, a Pearson’s two-tailed correlation was used once normality had been tested and met. To compare linear regressions of fluorescene data, analysis of covariance was used. To compare observational percentage occurrence data between surveys, a X^2^ test was used. All statistical analysis were conducted in Prism v. 5.0c. for Mac, GraphPad Software, San Diego, CA, USA. An alpha level of 0.05 was used for all statistical tests.

## Results

### Ecological surveys

**Figure 2:**
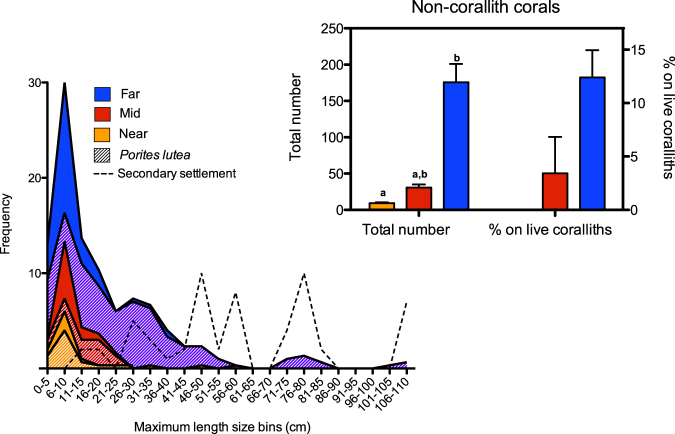
Size frequency distribution of coralliths using maximum length and 5 cm bins from triplicate 25 × 2 m belt transects 70 m (near), 140 m (mid), and 210 m (far) parallel from the seaward facing side of Vavvaru Island, Maldives. The contribution of *P. lutea* to the frequency of total coralliths is denoted with hatching. Dashed line indicates the minimum diameter of living coralliths observed to have secondary colonization by other corals (15 cm). The inset graph shows the total number of other (non-corallith) coral species along the transects on live coralliths, dead coralliths and large secondary rubble, with different letters indicating significantly different groups (Dunn’s Multiple Comparison, p = 0.03). The percentage of those that were found on living coralliths is also represented. Error bars represent standard error.

Coralliths were found at all transect sites, with varying sizes illustrating the progression from small coralliths to large, stable coralliths (Fig. 1). At all sites, the most common corallith size was 6–10 cm (Fig. 2); significantly more coralliths were observed within this size range at the far-shore site than either the mid- or near-shore sites (*F*_(2,6)_ = 5.29, *p* = 0.047). *P. lutea* coralliths accounted for ~73% of the total number of coralliths present (Fig. 2).

Both total corallith abundance and the frequency of larger coralliths (maximum lengths of >1 m) increased with distance from the shore (Fig. 2). *P. lutea* coralliths larger than 1 m were not observed in the sampling transects, but they were observed nearby; maximum dimensions of some were in excess of 3 m. The abundance of non-corallith forming species significantly increased from the near to far-shore site (*H* = 7.20, *p* < 0.05), and significantly correlated with the increasing abundance of coralliths (Pearson *r* = 0.93, *p* < 0.01, *r* ^2^ = 0.87). There was a significant positive correlation between the number of secondary species and corallith size (*r* = 0.73, *p* = 0.03, *r* ^2^ = 0.53). The percentage of corals found living on live coralliths significantly increased from 0% at the near-shore site to ~3.5%, and ~12% at the mid- and far-shore sites respectively (*χ* ^2^_2_ = 11.25, p = 0.004 between mid- and far-shore sites) (Fig. 2). Other corals observed were settled on either dead coralliths or on large rubble fragments. The minimum size of a corallith on which other corals settled was 15 cm (max. length), with multiple species being often found on coralliths over 30–45 cm (when bare skeleton was available for settlement; Fig. 2).

**Figure 3:**
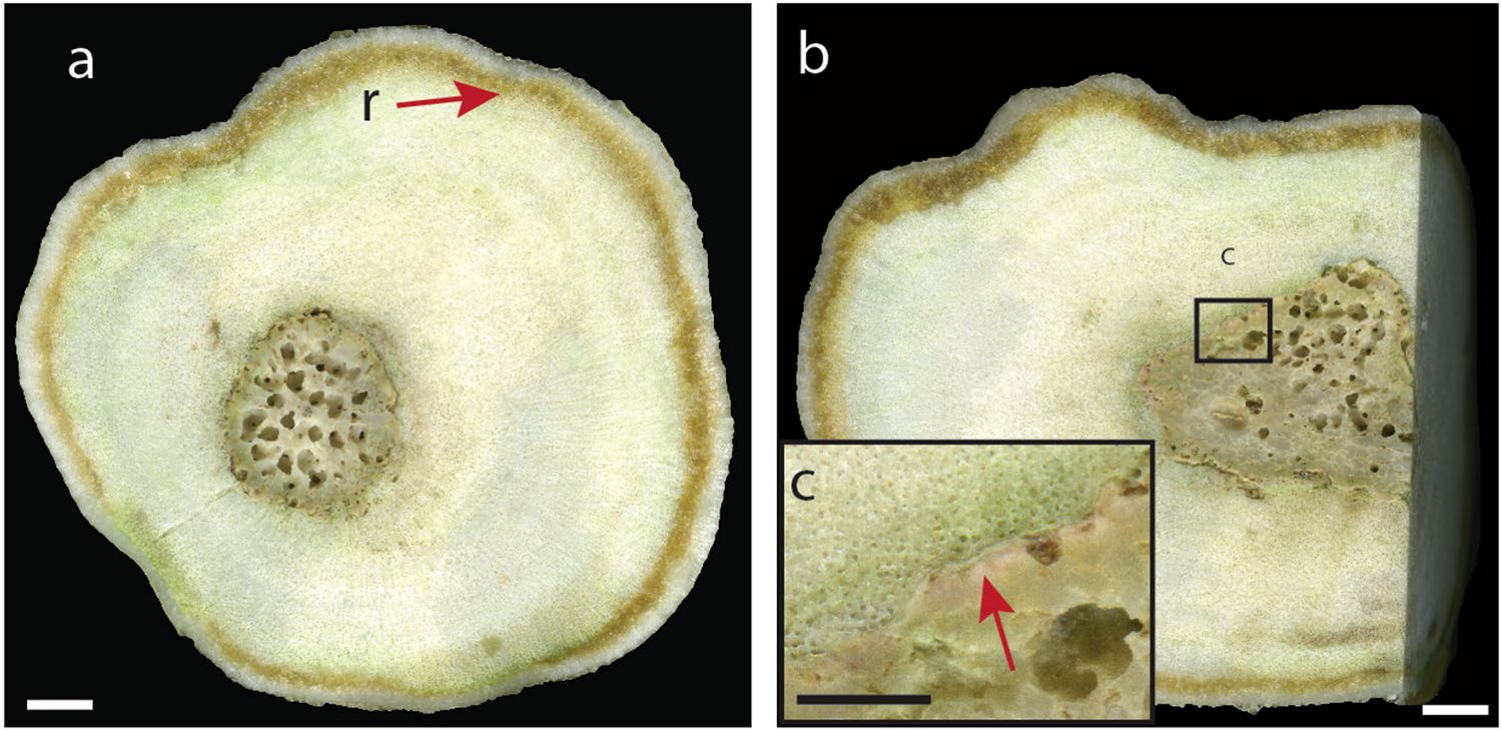
Cross (**a**) and longitudinal (**b**) section of massive *Porites lutea* corallith, grown around a branching coral fragment core. Brown line denoted by r is resin uptake by the coral skeleton during sample preparation. Inset (**c**) is a close up of the interface between the core coral fragment and the subsequent *P. lutea* colonization. The arrow in (**c**) indicates the presence of crustose coralline algae. Scale bar is 0.5 cm in (**a**) and (**b**), and 0.2 cm in (**c**).

The coralliths examined were mostly spherical or ellipsoidal in shape, reflecting the substrate they originally settled on (a small fragment or branch of coral), or the history the corallith had had in terms of periods of stability^5^. On sectioning the coralliths, CCA were observed at the interface between the corallith species and the base rubble (Fig. 3)^4^, which likely provided the crucial cues to promote coral larval settlement^25^.

### Physiological assessment

**Figure 4:**
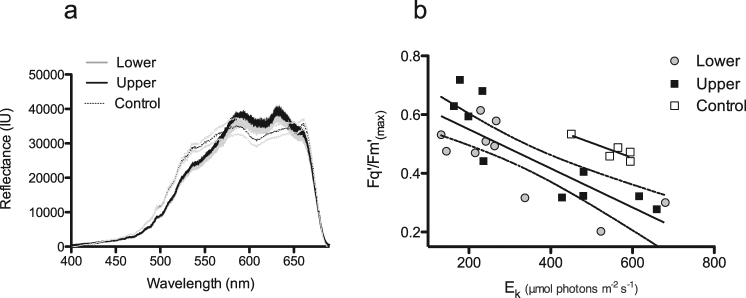
(**a**) Average reflectance profiles of the upper and lower surfaces of *Porites lutea* coralliths with shaded Standard Error (SE) (n = 10) from 400–680 nm compared to the top of static *P. lutea* controls (dotted SE for clarity, n = 5). (**b**) Derived maximum photochemical efficiency (Fq’/Fm’ _(max)_) and light saturation coefficient (E_K_) of coralliths and static *P. lutea* controls. Linear regression for combined upper and lower surfaces of coralliths (in figure with 95% confidence intervals), y = −0.0007x + 0.6828, r^2^ = 0.64, and for control corals, r^2^ = 0.74, y = −0.0005x + 0.7594.

To determine whether coralliths had unique physiology (compared to static controls) that may facilitate their dynamic life history, their photophysiology, O_2_ production, calcification rates, and reflectance were examined. Photophysiological parameters were measured on the upper and lower surfaces of coralliths (as found in the field), and compared to nearby static *P. lutea* colonies (which were attached to more typical coral substrate of exposed bedrock surface) to identify any characteristics that may be associated with corallith survival. The coralliths examined were visually and physiologically similar on both upper and lower surfaces (Fig. 4). Corallith reflectance profiles were similar on the upper and lower corallith surfaces (Fig. 4), although the static *P. lutea* corals had higher reflectance between 410 and 690 nm (Fig. 4).

Initial photosynthetic efficiency (*Fq’/Fm’*), and the light saturation coefficient, (E_K_), which describes the transition between light-limited and light-saturated photochemical efficiency, did not differ between static controls or the upper / lower surfaces of the coralliths (*H*_2_ = 0.91, p = 0.64), (*H*_2_ = 0.03, *p* = 0.98) (Fig. 4b). The derived maximum photosynthetic efficiency, *Fq’/Fm’*_(max)_ correlated significantly with E_K_ for coralliths (*r* = −0.68, *p* < 0.01), as described previously by Hennige *et al*.^19^ in the context of coral photoacclimatory potential across light gradients. The significant difference in linear regression intercepts between static controls and coralliths (ANCOVA; *F*_(3,40)_ = 5.71, *p* < 0.01) (Fig. 4b), highlights that at similar light saturation coefficients, *Fq’/Fm’*_(max)_ was lower in coralliths. The slopes from both linear regressions were not significantly different (ANCOVA; *F*_(3, 37)_ = 0.29, *p* < 0.83)

Oxygen production rates did not differ significantly between coralliths and static controls (0.61 ± 0.19; 1.73 ± 1.12 µmol O_2_ cm^−2^ h^−1^ respectively). Instantaneous calcification rates (alkalinity anomaly techniques) were also highly variable between individuals, and did not significantly differ (coralliths: −0.97 to 1.70 µmol CaCO_3_ cm^−2^ h^−1^; static controls: −0.08 to 1.54 µmol CaCO_3_ cm^−2^ h^−1^).

## Discussion

The common occurrence of *P. lutea* coralliths likely reflects the relatively hardy nature of the *Porites* genera in terms of environmental resilience^16^, acclimatory ability^23^, and its low aspect growth form of small, sunken polyps with robust corallite structure. While massive *Porites* sp. colonies can slowly colonize muddy or soft sediments by prograding^3^, this is the first time that their pivotal ecological role in forming stable habitat from free-living colonies (over annual-decadal timescales) has been documented. Crucially, the free-living stabilization hypothesis does not rely on gradual, iterative colonization from a nearby reef, so can be applied to soft sediments, rubble deposits, high-energy environments, and degraded reefs. The free-living stabilization hypothesis can also explain the spatially separated formation of coral reefs in otherwise uninhabitable areas for corals. This complements observations in the fossil record, where *Porites* sp. corals have been identified as pioneer colonizers of isolated patch reefs on soft sediment established during the Pleistocene^2^, indicating that coralliths, and in particular *Porites* sp., have been performing a fundamental role of habitat stabilization for tens of thousands of years. Other common corallith species identified in this study included *Psammocora haimeana* and *Cyphastrea chalcidicum*, both of which have also been noted to be relatively resilient to bleaching^16^, highlighting the resilient nature of coralliths. The shallow sites assessed here may also be exposed to periods of elevated temperature when tidal flushing is low, promoting the occurrences of coralliths able to withstand periods of higher temperature. Parrotfish grazing marks were noted on many coralliths, which will contribute to mobilization and turning.

Since atoll islands are dynamic in nature^26^ and in general migrate from the windward to leeward platform^27^, the three belt-transect zones used in this study also differ in terms of their exposure age, with the near-shore transect having the youngest exposure for coral settlement compared to the far-shore site. This is supported by the increasing presence of larger coralliths with increasing distance from the shore: at the far-shore site, the coralliths have had a longer period of time to grow, reach a critical mass, and support subsequent growth of other coral species. This would also explain why there are a greater total number of coralliths (across all size classes) at the far-shore site, as they have been exposed to many more coral recruitment events.

The critical mass for a corallith to become a stable coral colony will depend upon the strength of the local hydrodynamics, the initial substrate upon which settlement occurred, and environmental perturbations. In a low tide or wave energy system, ‘stability’ and lack of subsequent movement would be achieved with a much smaller mass or size than in a high energy system with frequent strong tidal surges or strong wave action. If initial settlement of the coral larvae was on a large irregular shaped rubble branch, then subsequent growth could also be irregular, providing stability compared to completely spherical coralliths. In the system examined here, critical size where movement decreased and the substrate-facing parts of the coral died (Fig. 1b) was a minimum of ~15 cm diameter, but was more commonly ~30 cm diameter. Once a corallith approaches its critical mass, its stationary residence time will increase, and at this point it could start to encrust surrounding substrate, or CCA from touching substrate could start to encrust any dead parts of the corallith. Successful encrusting would further increase the stability of the corallith. An interesting question is whether ocean acidification would reduce the efficiency of this process, as acidification could increase the erosion of suitable substrate^28,29^, and reduce the growth and development of reef-stabilising CCA^30^. This could increase the critical mass needed to become stable, as ‘secondary stabilisation’ through CCA or encrusting may be less efficient, and delay colonisation of the corallith by other coral species.

The increasing occurrence of non-corallith forming coral species with increasing distance from the shore is explained by an increase in the amount of settlement substrate over time as the coralliths grow and stabilize. Suitable substrate includes both living coralliths with dead patches (accounting for settlement of ~12% of non-corallith forming species at the far-shore site), and dead coral substrate. Dead coral substrate was noted to consist of dead coralliths which had reached critical mass before death, and degradation of previous generations of secondary settlement coral species, in many cases from growth on coralliths (alive and dead). Thus, once coralliths have reached a critical mass and created a stable substrate, settlement by other coral species enables self-perpetuating engineering of stable substrate for further corals to colonize.

Physiologically, coralliths were similar on their upper and lower surfaces (indicating regular turning), and did not differ significantly from static controls. While previous studies^19^ have documented consistent correlations between *Fq’/Fm’*_*(max)*_ and *Ek* across depths and between different species, coralliths from this study have a significantly different linear relationship than controls of the same species from the same light environment (Fig. 4b). The previous light histories of coralliths used in this study are unknown (i.e. how frequently they have been moving). Given that *Fq’/Fm’*_*max*_ is related to the ‘poise’ for non-photochemical quenching^19^, further investigation may reveal photoacclimatory ‘trade-offs’ between ensuring no photodamage occurs to symbionts on surfaces suddenly exposed to high light, and energetic investment into photoprotection mechanisms.

Different *in hospite* symbiont communities between coralliths and static controls may have influenced subtle differences in reflectance spectra^31^. The key to the success of free-living stabilization is for a coral to survive settlement on mobile substrate, to withstand mechanical damage from frequent movement, and be able to acclimate quickly to changes in environmental conditions, allowing growth to be maintained whilst minimizing cellular damage. Further studies are needed to investigate whether *Porites* sp. coralliths share the general environmental resilience of established *Porites* sp. colonies^32,33^, or whether they are less resilient to stressors such as temperature increases due to energy expenditure on tissue repair from abrasion.

Given their key ecological function, coralliths could play a role in conservation management, restoration, and policy practices. The use of artificial reefs and coral transplants in degraded areas are considered two major coral reef conservation strategies^34^. Whilst some success has been noted, these strategies have a number of documented problems, including failed coral attachment in new environments, high mortality rates, and depletion of corals from ‘donor reefs’^34^. As demonstrated here, coralliths can potentially naturally provide new, stable habitat over relatively rapid (decadal) timescales, and can support an increasing number of species as the coralliths grow. In places where no management systems are in place, coralliths may thus play an important role in continuous provision of new colonization substrate to promote coral reef growth.

### Data Availabilty

The datasets generated during the current study are available in the British Oceanographic Data Centre, https://www.bodc.ac.uk/data/published_data_library/catalogue/10.5285/4f8efa6e-3c92-5f3e-e053-6c86abc0b543/^35^).
